# Role of in vitro two-dimensional (2D) and three-dimensional (3D) cell culture systems for ADME-Tox screening in drug discovery and development: a comprehensive review

**DOI:** 10.5599/admet.1513

**Published:** 2022-10-10

**Authors:** Venkatesh Chunduri, Srinivas Maddi

**Affiliations:** Acubiosys Private Limited, Plot # D-14A, Industrial Area, TSSIC-IALA, Moula Ali, Hyderabad, Telangana 500040, India

**Keywords:** 2D In vitro, 3D in vitro, ADME, Toxicology, Organoids, Micro-physiological systems

## Abstract

Drug discovery and development have become a very time-consuming and expensive process. Preclinical animal models have become the gold standard for studying drug pharmacokinetic and toxicity parameters. However, the involvement of a huge number of animal subjects and inter-species pathophysiological variations between animals and humans has provoked a lot of debate, particularly because of ethical concerns. Although many efforts are being established by biotech and pharmaceutical companies for screening new chemical entities *in vitro* before preclinical trials, failures during clinical trials are still involved. Currently, a large number of two- dimensional (2D) in vitro assays have been developed and are being developed by researchers for the screening of compounds. Although these assays are helpful in screening a huge library of compounds and have shown perception, there is a significant lack in predicting human Absorption, Distribution, Metabolism, Excretion and Toxicology (ADME-Tox). As a result, these assays cannot completely replace animal models. The recent inventions in three-dimensional (3D) cell culture-based assays like organoids and micro-physiological systems have shown great potential alternative tools for predicting the compound pharmacokinetic and pharmacodynamic fate in humans. In this comprehensive review, we have summarized some of the most commonly used 2D in vitro assays and emphasized the achievements in next-generation 3D cell culture-based systems for predicting the compound ADME-Tox.

## Introduction

The current challenge in drug discovery programs for the pharmaceutical industry is increasing patient access to novel medications while reducing attrition rates. In recent times, despite many advances in disease knowledge and the drug discovery process, the translation of these benefits into therapeutic breakthroughs has been far slower than anticipated [1]. The major challenges a pharmaceutical industry faces during the drug discovery process include a high churning rate, duration of research, and changing regulatory considerations contributing to higher costs [2]. For a single drug, getting FDA approval requires an investment of US$1-2 billion and an average of 10-15 years of research and development [3]. Moreover, only one in every 5000-10000 candidates receives FDA approval. Thus, efforts have to be taken for alternate drug discovery and development processes [4]. Furthermore, these drugs can be expensive and linked with various side effects. The process of drug discovery includes three steps: 1. identification and synthesis of a new chemical entity; 2. preclinical trials, which include cell- and animal-based efficacy, toxicity, pharmacokinetic and pharmacodynamic profiles and 3. clinical trials. The major cause for the candidate drug failures is poor pharmacokinetic and pharmacodynamic profiles in Absorption, Distribution, Metabolism, Excretion (ADME) and toxicological (Tox) studies [5]. Thus, the evaluation of ADME-Tox mechanisms has become a fundamental step in the drug discovery process and selection of new drug candidates.

Presently, the best way to study the ADME-Tox processes is through *in vitro* and *in vivo* animal models [6]. However, because of inter-species variations, preclinical variations in toxicology findings between small (rodents) and large (dogs/monkeys) can’t replicate the exact mechanism of drug transport and metabolism as that of humans, as they have a more complex mechanism of cell-to-cell barrier and enzyme kinetics [7-9]. While animal models remain a requisite tool in ADME-Tox studies, non-animal models are a prerequisite for the transition towards animal-free qualification of new compounds [10]. *In vitro* models and cell-based assays have served as a basis for assessing the efficacy and risks of investigational novel drug candidates in humans. Two-dimensional (2D) *in vitro* cell culture systems use cells obtained from human tissues to mimic physiological and metabolic functionality. *In vitro* cell models were designed to simplify the methodology of the study of compounds that are cell or organ-specific. These models are relatively rapid and cost-effective and further help in indicating the ADME fate of the compounds [11]. However, the major drawback of using 2D cultures is that they can’t accurately illustrate and mimic the complex environment compared to *in vivo*, as their cellular organizations reflect their behaviour during culturing [12].

To overcome this, novel preclinical or para-clinical techniques must be developed to compensate for and elevate the use of better cell- and organ-based assays for a more precise representation of human response to drug candidates. Recently, three-dimensional (3D) cell cultures emerged as a promising tool for assessing drug absorption, drug disposition and pharmacokinetics (PKs), which influence drug safety and efficacy at an early stage in drug development. They have better recreation of tissue functionalities and provide more physiological relevant niches than 2D cultures, despite this, they continue to have an advantage over animal models. Presently, there are many different types of 3D cultures viz. spheroids, organoids, tumoroids, 3D models equipped with microfluid flow controls or micro physiological systems (MPS) and hybrid 3D systems that combine 2D culture with biomedical micro-electromechanical systems. As a result, when drugs are tested in 2D or 3D cultures, their effectiveness substantially changes, as 3D cultures are more resistant to drug treatments compared to 2D monolayers, which is analogous to what occurs in the human body [5]. This article reviews the current 2D cell line models used in ADME-tox screening and the applications and challenges of 3D organoids and MPS models in ADME tox studies.

## 2D cell culture systems to evaluate ADME-Tox screening

### Absorption

The major aim of the drug discovery program is to get a candidate that has a therapeutic effect at the site of action and is eliminated in a particular time frame [5]. Most drugs are designed for oral administration because it makes it easy for patients to self-administer. Drugs administered orally should pass through the gastrointestinal tract, be absorbed, and sent to the liver through the hepatic portal system, where it gets metabolized or distributed to the site of action. Absorption is the first biological process in the pharmacokinetic parameter of the drug, which mainly occurs at the intestinal level [13]. Hence during the drug discovery process, developing drugs that can efficiently penetrate the gut epithelium is critical. The absorption in the intestine is majorly hindered by the intestinal epithelial membrane composed of monolayer columnar cells comprising: 1. enterocytes are characterized by the presence of apical villi and microvilli over them, which regulates the diffusion of small compounds. They also produce a large amount of food and drug-metabolizing enzymes for exogenous substance protection. 2. Enteroendocrine cells, characterized by releasing peptide hormones, and 3. goblet cells produce mucous covering the intestinal epithelium. In fact, the mucous layer is the first barrier that filters nutrient absorption by maintaining intestinal homeostasis [14]. 4. Intestinal stem cells (Lgr5(+)) help maintain other cells' integrity and function by rapid dividing and differentiation. 5. Paneth cells, which help in providing support and secretion of growth factors and antimicrobial peptides [5]. The absorption of the drugs from the luminal side to mesenteric vessels on the basolateral side follows either: 1. Passive transport, via transcellular, i.e., passing through the cell membrane (mostly lipophilic) [15] or paracellular, i.e., passing through tight junctions or intercellular spaces (mostly hydrophilic) [16]. 2. Active transport, via influx and efflux through transporters present on the apical and basolateral side cell membrane or vesicular mediated endo- or trans-cytosis [17]. Cell-based models not only can mimic the *in vivo* anatomical, biochemical and structural features of the small intestine but also help in reproducing the study of both passive diffusion and active transport process [5]. To date, many immortalized cell cultures are used by researchers to predict the permeability and absorption of drugs. The absorption models will help us to determine the rate of transfer per unit area (J) which follows Fick’s law of diffusion and apparent permeability co-efficient (*P*_app_), which indicates the amount of drug passing the barrier per unit area (*A*). These are determined by using the following equations:


(1)

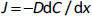




(2)

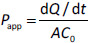



where *D* is diffusion coefficient (cm^2^/h), d*C* is the concentration gradient (g/cm^3^), d*x* is the linear distance traveled (cm), d*Q*/d*t* indicates the rate of drug transferred into the acceptor side and *C*_0_ is the initial drug concentration in the donor side [5].

### Artificial models for absorption

The most commonly used 2D *in vitro* models are synthetic or cell line based. Both these procedures have a donor plate comprising a permeable membrane or filter on which a synthetic phospholipid bilayer is attached, thus mimicking the intestinal niche. Synthetic models such as Parallel Artificial Membrane Permeability Assay (PAMPA) [18] and Phospholipid Vesicle-based Permeation Assay (PVPA) [19] are the cell-free permeation systems that help in reproducing the study of passive diffusion processes. Both these processes use an artificial membrane that mimics the phosphor-lipid bilayer of the intestinal enterocytes. The major difference between these two methods is in PAMPA, the phospho-lipid membrane is dissolved in an organic solvent, whereas in PVPA, it is organic solvent-free, and the structure is made up of a tight barrier comprising of liposomes between the pores and on the top of it is phospholipid bilayer [20]. Both these methods are considered best in assessing the absorptive potential of lipophilic drugs with high reproducibility, cost-effectiveness and easy handling compared to cell-based assays [21,22].

### Cell line models

#### Caco-2 (Human Colon Adenocarcinoma)

The most commonly used cell line model for studying the mechanism of intestinal absorption is Caco-2, derived from human colon adenocarcinoma. They are characterized by enterocytic phenotype having villi and microvilli as well as tight junctions after reaching full confluence [23]. The regulatory authority for ADME-Tox studies considers this model the gold standard as it provides absorbed and metabolized fractions of compounds across the intestinal epithelium [24]. Indeed, the Food and Drug Administration (FDA) recognized the Caco-2 model system as useful in classifying a compound’s absorption characteristics in the Biopharmaceutics Classification System [25]. The Caco-2 cell line model is considered efficient for studying the passive diffusion of lipophilic compounds due to its absorptive similarity with intestinal enterocytes [26]. Reports also suggest that the permeability coefficient of the Caco-2 model is correlated with *in vivo* fraction absorbed [27]. Although Caco-2 is considered a valuable tool for screening passively transported drugs, limitations were observed when we use the model for active transport due to the absence of P-glycoproteins (P-gp) [28] and multi-drug resistant (MDR) proteins [29]. Moreover, deficiency of some of the metabolizing enzymes such as cytochrome P450 3A4 (CYP3A4), absence of mucous layer and slow growth are also major drawbacks and significantly impact model accuracy [30-32]. In addition, the transmembrane resistance (TEER) is also higher (250-2500 Ω·*cm*2) as compared to the small intestine (12-120 Ω·*cm*2) [33].

#### TC7 (Cac-2 Clone)

To overcome some of the major limitations of the Caco-2 model, researchers have derived a TC7 cell line, which is isolated from Caco-2 subclones. Some major advantages of TC7 include faster growth, relatively lower TEER (150-750 Ω·*cm*2) and less heterogeneity [34]. Moreover, the number of metabolic enzymes at high confluencies, such as UDP-glucuronosyl transferases, hydrolase, sucrase-isomaltase and CYP3A isoenzymes, are also similar to that of the intestinal epithelium of the duodenum and jejunum [35]. In addition, the transcellular drug kinetics correlate with Caco-2, indicating TC7 can be an excellent option over Caco-2 [36,37]. Still, further investigations are required for large-scale evaluation to check the potential of the TC7 model.

#### MDCK (Madin-Darby Canine Kidney)

(MDCK is another cell line that can be used for permeability studies for its similarity with Caco-2 morphology consisting of brush border and tight junctions [23]. The major advantages are also lower TEER (173 Ω·*cm*2) and less growth period (3-5 days for full confluency), and subsequently, less cost [38]. Thus, the MDCK model can be used for studying the permeability screening of both intestinal and renal epithelia in the drug discovery process [38]. Still, there are some major limitations present in this model, which include: 1. intolerance to organic solvent, leading to less access to high lipophilic compounds; 2. non-intestinal origin and less homogenecity; 3. lack of transporters and metabolic enzymes [39]. To overcome this, researchers have transferred the MDR1 gene to induce its expression and observed overexpression of P-gps and increased digoxin efflux compared to its parent MDCK cell line [40]. However, in comparison with Caco-2, the MDCK model remains less applicable [41].

#### HT-29 (Human Colorectal Adenocarcinoma Cell line)

HT-29 is another cell line used for studying drug permeability by researchers. They are derived from human colon adenocarcinoma cells having characteristics similar to mature intestinal cells, like absorptive enterocytes and intestinal secretory cells that secrete mucous [42]. However, the differentiation of HT-29 cells is dependent on external media composition. Under the glucose-free condition, the HT-29 cells have characteristic similarities with intestinal epithelium like villi and micro-villi, tight junctions and metabolic enzymes associated with micro-villi [43]. Although HT-29 has characteristic similarity, it is less utilized due to the expression of hydrolases on the apical side and also the presence of several membrane receptors that are usually absent in the human intestinal epithelium. However, this model is most useful for studying the absorptivity of foods and xenobiotics due to its characteristic feature of producing mucus-like substances since it influences intestinal tissue homeostasis [44].

#### IEC (Intestinal Epithelial Cell line)

Rat IEC derived from rat ileal crypts is another model which gained importance in drug permeability studies [45]. Among different IEC subclones, IEC-18 is the most commonly used cell line. They develop confluent epithelium when grown on rat mesenchymal or similar tissue [46]. The characteristic features include the presence of tight junctions and desmosomes between the cells and microvilli on their apical surface. The lower TEER (28-55 Ω·*cm*2) in the IEC-18 indicates higher paracellular permeation than the Caco-2 model. Ma and colleagues [46] checked the paracellular hydrophilic markers like mannitol, dextran and PEG-4000 and observed greater permeability across IEC-18 than Colonic cells.

Human intestinal epithelial cells (HIEC) are another cell line gaining attention over Caco-2 and HT29 due to similar morphology to the *in vivo* intestine. The characteristic features include polarized columnar cells, mono-layer formation, and dense microvilli. In addition, the TEER value is 98.9 Ω·*cm*2 which is in a similar range to *in vivo* conditions due to its poor organization of tight junctions [47,48]. Takenaka and colleagues [49] evaluated the *P*_app_ and intestinal fraction absorbed (Fa) of polyethylene glycol oligomer compounds in the HIEC model and Caco-2. The results suggested that the HIEC monolayer had markedly higher precision to predict Fa values compared to the Caco-2 cells. Moreover, HIEC accurately classified 23 drug compounds having different Fa than Caco-2 [49].

### Distribution

After the drug absorption, it enters systemic circulation, from where the distribution process of the drug occurs. Distribution refers to the transfer of a drug from the intravascular (blood/plasma) and extravascular (intracellular & extracellular) compartments and vice versa of the body [50]. The drug distribution process is important because it can affect the amount of drug that ends up in the active sites and, thus, drug efficacy and toxicity. A drug will move from the absorption site to tissues around the body, such as brain tissue, fat, and muscle. The distribution process mainly occurs through systemic circulation, i.e., blood, and is influenced by blood flow, lipophilicity, molecular size, drug interaction with the blood components, like plasma proteins and partition/distribution coefficients. Researchers have developed several in vitro assays to assess the distribution process, like lipophilicity, solubility, plasma stability and plasma protein binding [11]. Lipophilicity gives the partition coefficient (log *P*) of the drugs between aqueous and non-aqueous phases under non-ionic conditions. It is also used for the determination of the distribution coefficient (log D) of the drug molecules in ionized forms. Since most of the drug molecules contain ionizable groups, they are likely to be charged at physiological pH (log *D* 7.4) [11]. Aqueous solubility measurement at physiological pH is another important parameter for estimating the distribution process. Poor soluble compounds will affect the ADME analysis, as a fraction of the compound gets precipitated, which may lead to the unavailability of the action cites.

The stability of drug compounds in plasma is another essential factor to assess the possible degradation or protein binding issues, as the blood plasma contains enzymes like hydrolases and esterases. These can affect both the approach and design of the bioanalytical assay as well as the *in vivo* outcomes. Plasma protein binding is assessed to determine the amount of freely available drugs in the plasma for distribution to the active sites. When the drug gets absorbed into the blood, the majority of the drug fraction binds to the plasma proteins, and subsequently, the amount of drug available to reach the target is reduced, thus determining the drug efficacy, metabolism and other pharmacokinetic parameters. In contrast, drugs that are less bound to plasma proteins are highly available for distribution to the organs and tissues, whereas drugs with high binding are only restricted to vascular spaces and have a very low volume of distribution.

### Metabolism

After the drug is absorbed through the intestine, it passes to the liver through the hepatic portal system, where most of the metabolism happens. The liver is the most important organ for drug development and pharmacokinetic studies. It helps in the elimination of many endogenous and exogenous substances from the body through drug-related transporters and phase-I and phase-II metabolizing enzymes [51]. Liver models are necessary for understanding the metabolic route, identification of metabolizing enzymes and their intermediates and final metabolites, transport, and other drug interactions, as it is responsible for the formation of drug metabolites and contribute to drug clearance and bioavailability [52]. These models are also necessary for understanding drug-induced liver injury (DILI), as most of the drug resides in the liver. To address this barrier issue in orally administered drugs, researchers have developed hepatic metabolic stability assays and hepatic metabolism by cytochrome P450 (CYP) enzymes. These assays are very useful in finding apparent clearance (*Cl*_app_), *in vitro* half-life (*t*_1/2_) for early screening and for ranking the drug candidates and revealing information about the possibility of the drug exposure variability due to metabolism by polymorphic enzymes, comparative metabolite fate between preclinical animals to that of human and also clinical interactions because of inhibition or induction [53].

The CYP450 family is the major target of the liver’s metabolic activity, which metabolizes a wide range of drugs. Among ~50 CYP isoforms, CYP3A4 accounts for around 50-60 % of all therapeutic drugs [54]. CYP inhibition assay has become an important determinant in the discovery and development of new drugs as they provide valuable information about drug exposure potential and metabolite fate among preclinical species to that of humans as well as clinical interactions caused by inhibition and induction [55]. Hepatic metabolism occurs in two phases. In phase-I, structural alterations in drug molecules occur, while in phase-II, the association of drugs with hydrophilic chemical moiety is mediated by various transferases such as UDP-glucuronosyltransferases (UGT) and GSTs, yielding the production of more polar metabolites. Phase-II metabolism is responsible for the detoxification of metabolites produced in phase I metabolism [56].

Other than CYP metabolism, several other *in vitro* models have been established for studying hepatic metabolism. These include primary hepatocytes, hepatic cell lines, precision-cut liver slices and microsomes. Due to limitations in the use of these models, such as loss of cell specificity and limited life span, primary hepatocyte cultures from different species become the preferred model for conducting *in vitro* toxicity and metabolic research [57,58].

#### Primary hepatocytes

Primary hepatocytes collected from different species like mouse rats and humans are widely used for studying *in vitro* hepatic clearance and *in vivo* prediction [59-61]. The major advantage of using this model is the availability of a full set of enzymes and cofactors in their physiological conditions [62,63]. Human hepatocytes have a distinct advantage, providing almost similar drug metabolic profiles *in vivo*. But the major limitations are the availability of liver donors, the disease status of the liver and person-to-person diversity. Moreover, individual variations in CYP expression and activity were already reported because of lifestyle differences, age group and hormonal status that affect the hepatocytes' activity [64].

Since freshly collected hepatocytes are difficult to obtain, several cryo-preservation methods have been developed by researchers for long-duration storage and supply without any changes in differentiated form or activity [65]. Sandwich culturing primary hepatocytes (SCH) between collagen matrixes is an alternative approach commonly used for assessing drug metabolism [66]. Compared to monolayers, SCH showed functional integrity, continuous release of albumin and stable CYP expression for long durations [66-68]. Moreover, these models resemble the *in vivo* conditions like the polarized expression of transporters in apical and basolateral membranes, thus providing a major advantage for studying transport and metabolism simultaneously [69]. Application of these SCH models includes: understanding the interactions between membrane transporters and metabolizing enzymes because they play a part in restricting a parent drug's systemic availability through metabolism, and efflux (Eg. GSTs and MRP2; CYP3A and P-gp) [70]. To understand the role of hepatic transporters on apical and basolateral sides during drug and metabolite disposal. For example, sodium-taurocholate co-transporting polypeptide (NTCP), organic cation transporter (OCT), and organic anion transporting polypeptides (Oatp) are present on the apical side for uptaking of drugs from the blood to the liver and multi-drug resistance (MDR) which are expressed on the basolateral membrane effluxes the drug or metabolites from the liver to the blood [71]. The other applications include elucidating the mechanism of DILI and hepatobiliary disposition [72].

#### Liver microsome, cytosols and S9 fraction

Metabolic stability assays by liver microsomes, cytosolic and/or S9 fractions are the most commonly used model for studying drug clearance *in vitro* apart from primary hepatocytes. Microsomes are the major repository of phase-I metabolizing enzymes, i.e., oxidation (majorly by CYPs), reduction and hydrolysis, which helps convert the lipophilic drugs into polar compounds by adding –OH or NH_2_ functional groups. The cytosolic fraction consists of various water-soluble metabolizing enzymes and involved phase-II metabolism, i.e., glucuronidation, methylation, acetylation, sulfation and conjugation, which helps in transforming the drug molecules or the metabolites from phase-I to more polar compounds ultimately making them easy excretion from the body. S9 fraction consists of both cytoplasmic and microsomal fractions, thus constituting both phase-I and phase-II metabolism. But the major drawback of this model is the lack of a cell membrane barrier that confronts the drug to reach the cytoplasmic compartment [63]. In contrast, the major advantage of this model is direct interactions of metabolizing enzymes with the drug make it suitable as a paradigm for screening studies [73].

#### Cell lines derived from hepatoma

The most frequently used cell lines include HepG2 or Hep3B, Huh7, and HepRG. The major advantages of these cell lines include accessibility, ease of use, stable phenotype and limitless potential for replication.

##### HepG2 (Liver hepatocellular carcinoma)

HepG2 is the most commonly used cell line for studying drug metabolism and hepatotoxicity studies. These cell lines are non-tumorogenic, having high proliferation rates and epithelial morphology having diverse hepatic functions. These cell lines are functionally characterized by the synthesis and secretion of plasma proteins, cholesterol and triglyceride metabolism, lipoprotein metabolism and transport, bile acid synthesis, glycogen synthesis, and insulin signalling [74]. But the major drawback of HepG2 is the limited expression of CYP enzymes. This very low expression is mainly due to the down-regulation of transcription factor CAR and PXR [75]. Even the expression of phase-II metabolizing enzymes such as uridine diphosphate glucuronosyltransferase, glutathione S-transferase (GST), sulfotransferase, or N-acetyltransferase (NAT) are also less when compared to primary hepatocytes, but their expression is more than CYPs. And finally, the expression of transporter proteins such as NTCP, bile salt export pump (Bsep), and Oatp are also very limited in HepG2 than primary hepatocytes [74].

##### HuH-7

HuH-7 cells, along with their derivatives HuH-7.5 and HuH-7.5.1, have been vastly used as a convenient substitute for primary hepatocytes [76]. These cell lines are also well-differentiated hepatoma cell lines which are functionally characterized by the secretion of albumin proteins and enzymes for carbohydrate metabolism [51]. Similar to HepG2, the use of these cell lines was restricted because of the limited expression of drug-metabolizing enzymes. But some recent reports revealed that upon DMSO treatment, these cells have well-differentiated morphology as that of hepatocytes, significant overexpression of drug-metabolizing enzymes and up-regulation of liver-specific proteins like albumin, transthyretin, HNF4a, and a1-antitrypsin [77]. Although there is an increased expression, the expression of prominent metabolism enzymes like CYP and UGT is 10-fold lower than that of primary hepatocytes [77]. Although the metabolizing enzyme activity is less in HuH7 cells, constant phase-I and phase-II enzyme activities were observed over the passages, making them the better alternative for HepG2 cells [78].

##### HepRG

These are established cell lines from chronic hepatitis C liver tumours. Studies have shown its bipotent capacity, which can differentiate into two phenotypes, i.e., hepatic type and biliary type [79]. Several studies have reported that the expression pattern of phase-I and Phase-II drug metabolizing enzymes is similar to that of primary hepatocytes under the presence of minimal DMSO concentrations. But their activity decreases once the DMSO is removed from the medium [80]. The major advantages of using this HepRG are less functional variation over the passages, morphological and functional similarity to that of primary hepatocytes under differentiated conditions, and no inter donor variation [51]. Moreover, these cell lines are susceptible to common CYP inhibitors. Studies reported that upon treatment with β-naphthoflavone, phenobarbital and rifampicin induced the expression of different CYP enzymes to multiple folds [81]. Reports also showed that HepRG is a better model for studying DILI. The response of HepRG towards acetaminophen, i.e., over-expression of genes related to liver damage, is similar to that of primary hepatocytes and much higher when compared to HepG2 cells [80]. This inductive capacity has made HepRG an excellent alternative model for primary hepatocytes. Moreover, the susceptibility of HepRG to various bioactivated toxins such as alphatoxin B1 makes them a better model for studying cytotoxicity studies over HepG2 cell lines [51].

### Excretion

Excretion is another important parameter in PK, as any drug absorbed should be eliminated from the body within a specified time. The liver is responsible for the biotransformation of absorbed drugs or exogenous compounds and helps in the storage and excretion of these compounds and their metabolites through bile. In addition to the liver, drug clearance is also contributed by various transporters and metabolizing enzymes in the GIT and kidneys [82,83]. Drug transporters are considered important determinants of drug accumulation within the cells and are often correlated with efficacy, drug toxicity, and drug-drug interactions. In addition to providing resistance to a wide range of drugs, transporters also play a major role in the ADME process [84]. These transporters are majorly classified into influx and efflux transporters. Influx transporters majorly belong to the solute carrier (SLC) family and are expressed in the plasma membrane of all organs. The SLC family mainly includes organic anion-transporting polypeptides (OATPs/SLCOs), organic anion transporters (OATs/SLC22As), organic cation transporter (OCTs/SLC22As), organic cation and carnitine transporters (OCTNs/SLC22As), peptide transporters (PEPTs/SLC15As). These transporters mediate the entry of drugs from blood vessels to tissues and organs either by passive diffusion or co-transport or active transport with the help of ATP hydrolysis. These SLC transporters are generally expressed on diverse tissue membranes like the intestine, kidney, brain and liver to show their therapeutic effect, toxicity, or metabolism in the case of hepatocytes.

Efflux transporters export the drug out of cells into blood vessels or excretory vessels by utilizing an ATP energy source. Most of the efflux transporters belong to ATP binding cassette (ABC) transporters family [85] except multidrug and toxin extrusions (MATEs/SLC47As), which belong to the SLC family. Among the ABC transporters, multidrug resistance (MDR), P-gp and BCRP are majorly involved in the excretion of drugs and their metabolites from hepatocytes to bile and renal epithelial cells to lumen. In the liver, these efflux transporters are expressed on basolateral and canalicular membranes of hepatocytes and play a major role in mediating the drugs and metabolites from hepatic cytosol to blood and bile [86]. Similarly, in the kidney, these efflux transporters are expressed on the apical membrane of the renal epithelial cells and play a major role in eliminating the drugs from epithelial cells to the lumen of the urine. Most renal transporters include OAT1, OAT3, OCT2, MATE, P-gp and BCRP. These transporters actively help in the excretion of xenobiotics from blood to urine. Researchers have developed various *in vitro* models like transporter assays and cell-based assays for determining the drug elimination process.

The *in vitro* models for studying the process of transport and excretion include membrane-based assays and cell-based assays. Membrane-based assays are generally used to identify the substrates and inhibitors of particular transporters. These assays can be performed by simple ATPase assays and membrane vesicular transporter assays. In the case of ATPase assays, the substrates or inhibitors are incubated with cells expressing ABC transporters and the generated inorganic phosphate is estimated calorimetrically [87]. Whereas membrane vesicular transport assay generally uses inside-out oriented membranes prepared from different cell lines expressing ABC (e.g., SF9 insect cells, HEK293 and MDCK cells) [88]. Compared to ATPase assays, vesicular assays have the upper hand as they can estimate the exact transport mechanism of a drug or inhibitor [89]. Van Staden and colleagues [82] investigated the drug candidates affecting transporter function and DILI using membrane vesicles prepared from hepatocytes containing a bile salt export pump, MRP.

Cell-based assays include the use of immortalized cell lines or primary isolated cells or recombinant cell lines expressing transporter proteins. The most commonly used cell lines are MDCKII and human embryonic kidney (HEK) 293 cells. The application of MDCK cell lines has already been provided in the absorption section.

HEK293 cells were developed by the transformation of HEK cells with adenovirus [90]. The primary advantage of the HEK293 cell line is the low expression of metabolic enzymes and endogenous transporters, which makes them an excellent source for the generation of recombinant cell lines which can express exogenous transporters [91]. Parvez and colleagues [92] stably generated recombinant HEK293 that can express OAT1, OAT3 and OCT1/2 transporters and studied the effect of 22 antituberculosis drugs on the uptake of para-aminohippurate, N-methyl-4-phenylpyridinium acetate and zidovudine. Their results demonstrated the inhibitory effects of several antituberculosis drugs on these transporters.

## 3D cell culture systems to evaluate ADME-Tox screening

Until now, most of the pre-clinical trials utilized commercial 2D cell lines and patient-derived xenograft models for screening compounds *in vitro*[93]. However, these models lack complex in vivo human environment niches and couldn’t give conclusive drug responses. Most of the drugs that passed pre-clinical trials have very few success rates in phase-II and III clinical trials because of a lack of effectiveness and safety [94]. To overcome limitations like drug efficacy and safety in 2D cell culture techniques, scientists and researchers were committed to developing drug screening models that are more effective, realistic, time-saving, and labour-saving for studying pharmacokinetics and pharmacodynamic processes [95]. The morphological and functional similarity between *in vivo* and the cell grown in 3D culture conditions have established 3D models as a valuable option. As a result, 3D models are considered to have a significant impact on drug screening, bridging the gap between cell cultures and animal models and potentially reducing the usage of animals in research [96]. The major pros and cons between the 2D and 3D cell culture models are tabulated in table 1. Organoids are self-organizing 3D cell cultures with a realistic microanatomy due to their *in vivo*-like self-organizing and self-renewing capacities [97]. They revolutionized tissue engineering by maintaining cellular complexity, which is comparable to that of native organs [98]. These 3D structures are generated by differentiated pluripotent stem cells (PSCs), primary adult tissues, tumours and fetal tissues [99]. Among them, human-induced PSCs (hiPSCs) are gaining much importance in disease research, generating disease-specific hiPSCs and as a source of continuous supply of human cells that are not often available [100].

### Intestinal models for drug absorption

Differentiating procedures for generating intestinal organoids or enteroids [101] and IECs [102] from hiPSCs were successfully reported. To date, many researchers generated hiPSCs-derived organoid cultures involving different organs of the gastrointestinal tract, such as the stomach [103], pancreas [104] and intestine [105,106]. Among them, enteroids are gaining much focus in studying drug permeability and pathophysiological mechanisms [5]. For studying intestinal drug absorption, an *in vitro*-grown enteroid needs to structurally and functionally resemble human intestinal epithelium and they should be cultured for longer periods [107]. Enteroids derived from hiPSC followed three different steps. The first is differentiation into definitive endodermal cells by Activin A [108], followed by differentiation into midgut and hindgut with the help of fibroblast growth factor (FGF4) and Wnt3a and finally, intestinal differentiation by using R-spondin1, noggin and human epidermal growth factor (EGF). In addition, the treatment with R-spondin1, noggin and EGF also proliferates human IECs [109].

Reports suggested that *in vitro*-generated enteroids replicated the *in vivo* tissue morphology and physiology like crypt villi and mucus secretion and showed functional activity such as CYP metabolizing activity for prolonged periods [110]. They are also characterized by a central hollow region containing differentiated cells like goblet cells, enteroendocrine cells, and enterocytes, which are extruded into the lumen forming villi- and microvilli-like structures and a crypt base where differentiated ISCs and Paneth cells reside. Typically, enteroids are generated either from primary tissues, such as isolated intestinal crypts or a single ISC expressing the Lgr5 marker [111-113] or from PSCs, such as hiPSCs and embryonic stem cells (ESC) [106,114]. These are further differentiated into other intestinal cell types, thus creating a multilineage culture system [115]. Although the enteroids have a perfect resemblance with the *in vivo* small intestines, their complex morphology and lack of other supporting cell types, such as epithelium-lined blood vessels and immune cells, may constitute significant drawbacks for studying the drug transport, pharmacokinetic analysis and disease modelling [116]. Their closed lumens limit access to the apical surface, hindering the drug absorption studies and coculturing of pathogens [116,117]. It is challenging to perform quantitative assessments such as paracellular and transcellular assays without changing the organoid structure [118]. The microinjection technique was found to be promising for accessing the luminal side for studying the absorption of drugs, food and toxins across the intestinal epithelium [116]. Several reports are available that use microinjection techniques for studying the absorption of different molecules, such as monosaccharides and peptides, with the help of fluorescent tracers [119,120]. However, the application of this technique is limited due to expensive fluorophores, less reproducibility and irreversible damage caused during microinjection [120,121]. To overcome such limitations, researchers tried to adopt other techniques, where the 3D organoids are mechanically disrupted and then replated onto 2D plates for recovery, thus allowing the utilization of both apical and basolateral sides [122]. The organoids that are generated from hiPSCs were mechanically separated and filtered by the mesenchymal cells to avoid pharmacokinetic function. The purified cells were replated and grown as a hiPSC-IEC monolayer. This hiPSC-IEC monolayer was further used for screening metabolites that have specificity for CYP3A, which cannot be reproduced by conventional 2D models. The model was demonstrated to be more reliable in assessing the uptake of other molecules, proving the dependability of organoid-derived IEC monolayers for evaluating xenobiotics absorption. However, mechanical disruption may cause irreversible damage to the stem-cell compartment, thus affecting the propagation and differentiation of the organoid. The authors also stated much refinement in this technique is required to ensure reproducibility and replicability to perform thigh throughput assays. Thus enteroids may not be the best tool for evaluating the drug absorption processes through the intestinal barrier. Further, the lack of a vascular mimicking system, that helps in the transport of nutrients and waste also impacts the reliability of this model. To overcome this, alternative strategies like integrating these enteroids with dynamic flow systems like bioreactors and miniature flow chips where precise physical, biological and chemical conditions might be imposed [123-125]. In this, hiPSC and tissue engineering techniques generated tube-shaped epithelia, which they named mini-intestine containing crypt- and villi-like domains with an accessible lumen. The structure has a much similar spatial arrangement as that of *in vivo*. When this tube structure was connected to an external pump, the miniguts were penetrable, which allowed constant elimination of dead cells, thus prolonging its life span for several weeks. These structures also helped in the co-culturing of microorganisms for modelling host-microorganism interactions. The mini-intestines have all the cell types often found in conventional organoids. These structures preserve important physiological characteristics of the intestines along with regeneration potential. In addition, the researchers stated that the concept of generating the functional organ-on-a-chip models attains physiologically relevant organoid shapes, size and function along with very broad application in drug discovery. Pérez-González and colleagues[126] developed mechanically accessible intestinal organoids on the hydrogels. They observed lapse force mapping with sub-cellular resolution on their organoid monolayer, which provided several advantages over conventional organoids that are generated over an extracellular matrix. These include easy optical access, flexible mechanical environment management, and an open lumen that more closely resembles the open-tube shape of the intestine and eliminates the accumulation of dead cells. In addition, these organoids displayed a collective migratory pattern from the crypt to the villus that is not often observed in conventional organoids. However, their models have shorter transit amplifying zone, irregular geometry of the villus, thinner cells in the villi and lack of a well-developed brush-boarder compared to *in vivo*. Similarly, Yang and colleagues[127] also developed intestinal organoids from isolated crypts of the murine small intestines. They demonstrated the crypt morphogenesis in intestinal organoids by increasing the cell volume of differentiated enterocytes resulting in a pressurized lumen. Nevertheless, the generation and maintenance of miniguts are much more challenging and expensive for consideration in pre-clinical trials. Even the outcomes derived from drug toxicity and efficacy studies using these models often lack reproducibility due to the intrinsic variability of the organoid source, shape and size [128]. All these factors hamper the translational potential of miniguts in obtaining robust statistical results in PK profiles of the novel drug entities [5,129]. Hence further efforts are required in the development of cost-effective and much feasible organotypic research by considering its potentiality and reducing animal testing in drug discovery and development [130].

### Liver models for drug metabolism

Currently, the majority of the studies use 2D *in vitro* models and animal models for understanding drug metabolism; however, the complexity of human *in vivo* and interspecies variation results in variations in the actual prediction of drug metabolism. Further, these animal models are unsuitable for high throughput screening of small-library molecules for identifying drugs for disease treatment [131]. But can be achieved by using 2D hiPSC-derived hepatocytes platforms [132-134]. Small drug molecules/drug libraries aimed at the attenuation or reversion of the effects of diseases like alpha-1-antitrypsin (AAT) deficiency [135], familial hypercholesterolemia [136], and mitochondrial DNA depletion syndrome (MTDPS3) [137] have been achieved by using hiPSC derived hepatocytes platforms. Similarly, these platforms were also used for toxicity assessment of test compounds known to be toxic and non-toxic for cell morphology and viability [138-140]. However, all these studies are mainly focused on hepatocytes, but as we know that the liver is a complex organ consisting of different cell types like parenchymal cells, which include hepatocytes and cholangiocytes and non-parenchymal cells, including Kupffer cells, liver sinusoidal endothelial cells (LSEC), hepatic stellate cells, liver infiltrating lymphocytes [141,142]. None have included the non-parenchymal cells, which hold great importance in liver physiology. For example, LSECs are involved in most liver diseases, making them an attractive therapeutic target [143]. Moreover, Kupffer cells also played a crucial role in DILI and other liver diseases and were not used for drug screening and metabolism.

Although primary human hepatocytes are regarded as the gold standard model for determining hepatic metabolism, the decline in proliferative potential and long-term functionality limited its studies *in vitro* [144]. To overcome the limitations of 2D hepatocytes cultures and to achieve reduced animal usage in drug discovery programs, various researchers developed alternative 3D models utilizing hiPSC and generated liver-on-chip or MPS [145] and organoids [146,147] for studying the drug metabolism, DDI, clearance and bioavailability [148]. Currently, the most challenging aspect is the generation of physiologically and pathologically similar hepatic models to that of the functional human liver and maintaining cellular viability for several days. Considering these, there is an urgent requirement for the development of robust protocols for generating liver organoids or liver MPS in a scalable and miniature fashion.

The development of 3D liver models is becoming increasingly important in drug discovery, as they can replicate biological aspects like spatial arrangement, cell-cell and cell–ECM interactions [141]. Researchers have developed various protocols for the generation of organoids, and most prominently, the use of hiPSC has enabled the generation of more complex organoids containing different cell types of the organ [142]. Reports citing the generation of liver organoids from hiPSC are increasing daily [147,149-151]. Initially, the liver organoids were generated from the co-culturing of different cell types. Later advanced techniques were developed for the generation of organoids from the homogenous population [147,152,153]. Takebe and colleagues [149] have generated liver bud organoids by mixing the hepatic endodermal cells generated from hiPSC, with human umbilical vein endothelial cells and human mesenchymal stem cells. For maturation of these bud organoids, they supplemented the medium with an endothelial growth medium resulting in improved functionality. More recently, the same group has also developed complex organoids containing hepatocytes, endothelial cells and STM from hiPSC [150]. A similar approach has been utilized by Pettinato and colleagues [151] and cocultured hiPSC along with human adipose microvascular endothelial cells for the generation of organoids. They demonstrated that their organoids contain 89 % albumin+ and 15 % CD31+ cells and improved human hepatic functions similar to *in vivo* liver cells.

Liver organoids were generated from the differentiated hepatoblasts with the addition of EGF [152]. These organoids contain both hepatocytes and cholangiocytes. Similarly, Wu and colleagues [153] also generated a protocol through which the generated liver organoids contain 60 % albumin+ hepatocytes and about 30 % CK19+ cholangiocytes. Recently, a group of researchers has developed a robust protocol for the generation of liver organoids containing hepatocytes, HSCs, Kupffer cells, and cholangiocytes from hiPSC [154]. They first differentiated hPSCs to foregut spheroids liberated from the 2D culture and embedded them in Matrigel. The addition of retinoic acid helped in further differentiation into both parenchymal and non-parenchymal liver cells [154]. Similarly, Mun and colleagues [147] developed a protocol for the generation of liver organoids. They demonstrated that their protocol was reproducible and took relatively less time to generate organoids. The organoids are scalable, self-renewed, and showed rapid proliferation and maturation. Moreover, they also showed liver functional properties after 20 passages and good viability after cryo-preservation [147].

Other than organoids, drug screening studies showed that liver-on-chip or liver-MPS demonstrated significant potential for drug PKs and toxicity research [155,156,157]. In liver-MPS, cells are continuously grown in perfused chambers to achieve in vivo liver physiological functions [158]. Although there has been significant progress in the development of liver-MPS, however, the majority of the researchers used 2D hepatocytes for developing liver MPS. A reliable 3D *in vitro* model with a stable phenotype that can maintain morphology, viability and hepatocyte-specific function for a prolonged period still needs to be developed. Researchers are putting efforts into achieving this. Liver-MPS developed by Bavli and colleagues [159] reported maintaining stable physiological conditions for one month with real-time monitoring of mitochondrial respiration. They also measured the glucose and lactate electrochemically using computer controlled microfluid switchboard. Jang and colleagues [160] developed rat, dog and human liver-MPS models for exploring hepatotoxicity safety testing, drug action mechanism, and biomarker identification. Their finding implies that species-specific liver-chip can be used to predict species-specific hepatotoxicities and are further helpful in assessing the risk of drug-induced liver toxicities in humans as found in animal studies. These models may also be utilized to determine human hepatotoxicities and mechanisms of action. Recently, researchers developed a novel liver-MPS model and could able to achieve differentiation of hiPSC into organoids inside the chip [161]. These organoids were characterized by both hepatocytes and cholangiocytes, along with increased cell viability and maturity. Moreover, the organoids generated in this liver-MPS platform have shown high CYP enzyme expression and acetaminophen-mediated dose-and time-dependent toxicity responses. All these results suggest that MPS technology constitutes a valid platform for drug testing.

### Organoid models to evaluate drug toxicity

Drug-induced toxicity during the drug discovery process may lead to the discontinuation of research programs [162]. Drug-induced toxicity can occur in various organs and tissues in the body and causes acute injury [163]. Although animal studies are being conducted to predict the risk of drug-induced toxicity, because of species variation, only 40-50 % toxicity can be predicted [164]. Consequently, many researchers have developed *in vitro* 2D cell-based assays to predict drug-induced toxicity. However, these assays utilize single-cell types and cannot recapitulate the physiological organ function due to the absence of different cell types. The development of organoids has shown great promise for studying drug toxicity evaluations as they are more anatomically and functionally close to the organs in the living body [165].

The most common drug-induced toxicity is DILI, as the liver is the first-pass organ and where most of the drug metabolism occurs. To date, DILI has been evaluated using PHH or hepatocytes derived from hiPSC. But these assays couldn’t recapitulate physiological liver function since, in addition to hepatocytes, the liver is also composed of several other cell types such as cholangiocytes, stellate cells, Kupffer cells, and LSECs. To overcome this, researchers succeeded in developing liver organoids consisting of a more physiological similar *in vitro* hepatic model and performed toxicity assessments. Liver organoids developed by Sgodda and colleagues [166] from embryonic stem cells (ESC) were more sensitive to acetaminophen-induced toxicity than 2D cultured ESC-derived hepatic cells. Forsythe and colleagues [167] generated liver organoids composed of 80 % hepatocytes, 10 % hepatic stellate cells and 10 % Kupffer cells and evaluated four environmental heavy metals. They observed dose-dependent toxicity and demonstrated the use of 3D organoids in toxicity assessments. Similarly, a comparative assessment of three marketed phospholipidosis drugs on 2D HepG2 cells and 3D liver organoids showed that the organoids are more sensitive to drug-induced phospholipidosis [168]. Recently, Shinozawa and colleagues [169] evaluated high throughput toxicity screening of 238 compounds, which include 206 DILI compounds using bile acid transport activity and cell viability assay. The results showed 88.7% sensitivity and 88.9% specificity. They also demonstrated that CYP2C9*2 HLO is involved in bosentan-induced cholestasis, suggesting that by employing liver organoids, various susceptibilities dependent on the polymorphism can be obtained.

Drug-induced cardiotoxicity (DICT) is also one of the major causes of attrition during the drug development process [170]. Many drugs have been reported to cause prolonged QT and ventricular arrhythmia [171]. Moreover, most cancer-related drugs are reported to cause a reduction in the left ventricular ejection fraction (LVEF) and chronic heart failure upon long-term medication [172]. To assess the DICT, ECG is used to monitor the cardiac functions during the pre-clinical trials in both rodents and large animals. To reduce animal usage, preliminary *in vitro* assays have been developed, such as binding assay in HEK293 cells that express human ether-agogo related gene (HERG) K+ channels [173], which has been widely used to identify compounds with high QT. Recent advances in iPSC-derived cardiomyocytes [CMs] have also become increasingly utilized tools to predict drug-induced QT and arrhythmia [174-177]. However, these assays are only specific to CMs, but the role of non-CMs, such as primary human cardiac fibroblasts, cannot be neglected in studying the DICT [170]. 3D human cardiac organoids have gained importance as they mimic human cardiac physiological characteristics *in vitro*. Several researchers have developed 3D cardiac organoids by assembling cell suspensions composed of PSC-derived CMs and various non-CMs are seeded in hydrogel molds with ECM [178-181]. Cardiac organoids generated through this approach have shown better similarity to the human heart and can be useful for disease modelling, regenerative medicine and drug development [170]. In another approach, researchers implanted suspensions of both hiPSC-derived CMs and cardiac fibroblasts in hydrogels and cultivated them on a BiowireTM II platform for the generation of organoids. This platform helps record cardiac contractions, action potentials and conduct velocities [182]. A similar approach has been used to study the effect of various inotropes on cardiac contractility and observed good concordance, suggesting the broad application of this assay platform in the drug discovery process [183,184]. In another study, researchers developed 3D electromechanically coupled *in vitro* fluid pumping chambers that mimic ventricular contractions [185,186]. Using this engineered technology, they were able to measure the cardiac output and stroke volume for 25 cardioactive compounds and achieved 80-100% accuracy in assay prediction. Archer and colleagues [187] have developed organoids consisting of CMs, cardiac fibroblasts and cardiac microvascular endothelial cells. Using these organoids, they were able to measure different parameters like ATP depletion, mitochondrial membrane potential and endoplasmic reticulum integrity for 29 FDA-approved cardiotoxins. Interestingly, using cardiac organoids post-myocardial infractions were also modelled *in vitro* and reported doxorubicin-cardiotoxicity [188]. However, the cardiac organoids reported till now don’t contain all the cell types present in the adult human heart, such as immune cells, which protect against inflammation. Thus, novel technologies have to be implemented, such as co-culturing with immune cells may help gain knowledge on interactions between CMs and immune cells. Another drawback with cardiac organoids is the degree of maturation of different cell types is not similar to that of adult human tissue. Efforts toward maturation will help in toxicity prediction more accurately [189].

Drug-induced kidney injury (DIKI) is also another problem causing almost 19% of acute renal failures worldwide [190]. Drugs like cisplatin, NSAIDs, and antibiotics cause DIKI [191]. There is no ideal 2D *in vitro* assays available for studying the DIKI. To date, DIKI is predicted by using only human renal proximal tubule epithelial cells (RPTE) *in vitro* [192]. However, this model predicts almost 80 % of DIKI. But the kidney is a complex organ consisting of more than 20 different cell types having variable toxicity with different nephrotoxic compounds. To address these issues, researchers started developing 3D-based human renal tissue systems. The 3D systems developed using human renal cortical NKi-2 cells showed more sensitivity to cisplatin, doxorubicin and gentamicin than 2D adherent NKi-2 cells [193]. 3D kidney organoids developed from hiPSC contain proximal tubules, podocytes and endothelial cells [194]. These organoids showed overexpression of Kim-1 upon treatment with cisplatin and gentamycin. Czerniecki and colleagues [195] developed a high throughput screening platform for the generation of kidney organoids from hiPSC and performed toxicity analysis for polycystic kidney disease. King and colleagues [196] developed 3D proximal tubule tissue consisting of RPTE, renal fibroblasts and endothelial cells using a 3D bioprinting platform and observed the formation of tight junctions, elevated renal uptake and efflux transporter levels and polarized localization and function of P-gp and SGLT2. These organoids showed dose-dependent cisplatin toxicity and rescuing effects of cimetidine, thus confirming the role of OCT2 transporter in cisplatin-mediated toxicity. To date, all the studies on kidney organoids have targeted RPTE. However, because of the complex nature of the kidney, it is also important to develop organoids consisting of glomerulus cells and prediction of glomerular toxicity. Further, more efforts are required to create innovative *in vitro* assay techniques that more accurately depict the physiological processes of the human kidney.

Drug-induced neurotoxicity is a serious problem causing both tissue damage and functional impairment, such as seizures, leading to drug attrition [197]. Previously, preclinical neurotoxicity assays were predicted only by using *in vivo* models. After the discovery of hiPSC and the development of methods for neuronal cell differentiation, *in vitro* toxicity assays were developed to study neuronal toxicity using hiPSC-derived neurons. The commonly used 2D hiPSC-derived neurons *in vitro* assays include the determination of neuronal death neurite outgrowth, calcium oscillation, and extracellular field potentials [198-201]. However, the brain is the most complex organ there is a requirement for understanding the structural and functional neurotoxicity. The creation of organoids has opened up the scope of understanding the drug effects on different parts of brain tissues. Moreover, brain organoids are predicted to provide vital information to increase our understanding of the mechanisms of neurodevelopmental or neurodegenerative disorders, as well as useful tools for disease modelling and drug screening [202]. The first report of 3D self-organizing cerebral organoids was reported by embedding the neuroectoderm derived from human stem cell-induced embryoid bodies onto matrix gel [203]. When these embryoid bodies were transferred to a spinning bioreactor, they formed self-organizing cerebral organoids. Further, changing the composition of morphogen during the culture, these organoids were differentiated into different brain tissues such as the cerebral cortex [204], hippocampus [205], cerebellum [206] and midbrain [207]. Most of these hiPSC-derived organoids have similarities to the fetal brain studies that have been conducted to understand the mechanism of fetal alcohol spectrum disorders [208]. These ethanol-exposed cerebral organoids were found to have increased caspase-3 activity and altered the morphology and function of mitochondria. They also observed that the alcohol induction cases more apoptosis in neurons than astrocytes. Similarly, brain organoids exposed to acrylamide (a common food contaminant) cause a significant increase in nuclear factor erythroid 2-related factor 2 (NRF2)-mediated gene expression, induction of cell apoptosis, repression of neuronal differentiation, and promotion of tau hyperphosphorylation [209]. Neurotoxicity assessment of vincristine on brain organoids showed dose-dependent neurotoxicity and inhibition of fibronectin [210]. Pamies and colleagues [211] have generated cerebral organoids consisting of mature neurons and glial cells and studied the effect of rotenone. They observed the toxicity of rotenone varied on the differentiation status of the organoids. These reports suggested the scope of 3D organoids in understanding the screening of drugs for neurotoxicity. However, there is a requirement for the development of more advanced cerebral organoids as there are several blood barriers involved *in vivo*, such as tight junctions, nutrient transporters, and increased expression of transendothelial electrical resistance.

## Conclusion

Previously, ADME-Tox screening has relied on preclinical *in vivo* models, but because of inter-species variations in physiology and functions, the data obtained can’t accurately recapitulate the mechanism of the ADME-Tox. Moreover, the preclinical animal models are expensive and time-consuming and can’t be used for high throughput screening particularly because of ethical concerns. Therefore *in vitro* assays and technologies utilizing human cells are required to overcome these limitations and to align the concept of the 3Rs (replacement, reduction and refinement). In this review, we summarized the available 2D and 3D *in vitro* predictive models for assessing the ADME-Tox screening. Preclinical 2D *in vitro* cell models are necessary for the screening of a large number of molecules in a cost-effective manner. Although the primary aim of 2D *in vitro* assays is to provide the prediction of compound fate and estimate the dose *in vivo* equivalent, still there are a large number of compounds getting attrition from research programs because of complex physiology. 3D *in vitro* cell-based assays have provided scope for studying the ADEM-Tox processes because of the physiological similarity to that of *in vivo*. Majorly organoids showed potential usefulness in toxicology studies, whereas MPS showed potential in studying the ADME process. Still, there is a requirement for improving the 3D *in vitro* models to mimic more physiologically relevant human organs. Further, there is a large scope in the utilization of MPS technology where multiple organ-on-chip models can be interlinked, which is further helpful in studying the mechanism of organ cross-talk [212-214]. These initiatives will enable a better understanding of highly predictive assays for ADME-Tox, and the selection of superior drug candidates during the development process.

## Figures and Tables

**Table 1.** A comparison between 2D and 3D in vitro models for ADME-Tox screening

Model	Pros	Cons
2D-Cell cultures	Ease of handling, interpretation and manipulation. Less training required. Economical. Can be used for high throughput screening. Less inter-lab variation. Reproducibility. Long-term storage.	Mostly cancerous origin. Less physiological relevance and lack of complex *in vivo* niches. Absence or limited production of enzymes and transporters. Not capable of mimicking the *in vivo* anatomical, biochemical and structural features. Poor drug metabolism. Can’t predict conclusively *in vivo* ADME-Tox properties.
3D Organoids	Self-organized and physiological similarity to that of *in vivo* tissues and organs. Ability for indefinite expansion and storage. Reproduction of cellular heterogeneity and cell-cell interactions. Absence of cancerous cells. High expression of tissue-related enzymes and transporters. Can be used for high-throughput screening. Similar gene expression profiles as that of *in vivo*. Disease modeling and development of disease markers. Drug screening for personalized therapy in case of cancerous patients.	Handling expertise is required. Expensive. Complex experimental techniques. Culture formation is time-consuming.
3D Micro-Physiological Systems	Physiological environment similarity. Can be developed using either 2D cell cultures or 3D organoids. Able to achieve physiological fluid flow levels and shear forces Can be used to develop multi-organ chip models. Conclusive prediction of ADME-Tox fate of a compound.	Complex fabrication process for model development. Sophisticated environment is required. Highly skilled expertise. Very expensive. Less scalable.
